# The Role of Prior Knowledge and Intelligence in Gaining from a Training on Proportional Reasoning

**DOI:** 10.3390/jintelligence10020031

**Published:** 2022-05-25

**Authors:** Christian Thurn, Daniela Nussbaumer, Ralph Schumacher, Elsbeth Stern

**Affiliations:** 1 Chair for Research on Learning and Instruction, ETH Zürich, Clausiusstrasse 59, 8092 Zürich, Switzerland; elsbeth.stern@ifv.gess.ethz.ch; 2 Institute for Special Learning Needs, University of Applied Sciences in Special Needs Education, 8050 Zürich, Switzerland; daniela.nussbaumer@hfh.ch; 3 MINT Learning Center, ETH Zürich, Clausiusstrasse 59, 8092 Zürich, Switzerland; ralph.schumacher@ifv.gess.ethz.ch

**Keywords:** proportional reasoning, prior knowledge, intelligence and transfer, physics learning, preparation for future learning, mediation model

## Abstract

We explored the mediating role of prior knowledge on the relation between intelligence and learning proportional reasoning. What students gain from formal instruction may depend on their intelligence, as well as on prior encounters with proportional concepts. We investigated whether a basic curriculum unit on the concept of density promoted students’ learning in a training on proportional reasoning. A 2 × 2 design with the factors basic curriculum unit (with, without) and intervention context to introduce proportional reasoning (speed, density) was applied in two consecutive, randomized classroom studies (N1 = 251, N2 = 566 fourth- and fifth-graders; 49%/56% female). We controlled for intelligence and mathematical achievement. We expected the combination of having received the basic curriculum unit on floating and sinking and proportional reasoning introduced via density (a familiar problem-solving context for this group) to be especially favorable. Whereas this hypothesis was not supported, we showed that mathematical achievement mediated the relation between intelligence and proportional reasoning and enabled learners to better exploit the learning opportunities.

## 1. Introduction

The period from late childhood to adolescence is one of great change, not only in executive control and emotional regulation but also in terms of universal, as well as differential aspects of cognition (Demetriou et al. 2005). Universal changes concern competencies, formerly considered as markers for reaching the formal operational stage according to Piaget: deductive reasoning (Halford and Andrews 2004), hypothesis testing by control-of-variable strategies (Schwichow et al. 2016), and proportional reasoning (Tourniaire and Pulos 1985). Expanding on the example of proportional reasoning, we want to shed light on the interaction between individual cognitive abilities measured with an intelligence test, prior knowledge, and the use of learning opportunities at two different time points. The goal of our study is to research the relations between intelligence and prior knowledge when exploiting training on proportional reasoning. The results presented here are based on the Swiss MINT Study (see Edelsbrunner et al. 2018; Schalk et al. 2019), a longitudinal study that investigated the effects of early physics curricula in elementary school (MINT is the German acronym for Mathematics, Informatics, Natural Science and Technology; STEM in English). In the Swiss MINT Study, elementary school teachers were trained in implementing physics curricula. A central, basic curriculum unit dealt with the floating and sinking of objects in water, and, in an inquiry-based manner students were supported in developing a basic understanding of the proportional concept of density. In the present quasi-experimental study, a portion of the students underwent this basic curriculum unit while others did not. Later on, all students received three training lessons on proportional reasoning. Therefore, this study allows us to disentangle the impact of conceptual knowledge about density, of mathematical achievement acquired throughout elementary school, and of general intelligence on gaining from training on proportional reasoning. With this design, we investigate whether undergoing a basic curriculum unit on floating and sinking, which, incidentally, conveys concepts fundamental for the development of proportional reasoning, prepares students for future learning of proportionality by transferring in (Schwartz et al. 2005). As we collect measures of intelligence and mathematical achievement from all students, we also investigate whether and to what extent individual differences in prior knowledge mediate the use of intelligence when undergoing formal instruction.

### 1.1. Attempts to Disentangle the Intertwined Impact of Prior Knowledge and Intelligence on Future Learning 

Many studies revealed strong effects of prior knowledge on future achievement (e.g., Holmes 2014; Simonsmeier et al. 2021; Weinert et al. 1989), a result that was retained when controlling for general cognitive abilities (Siegler et al. 2012; Stern 2009). Such results do not call into question the impact of general cognitive abilities on learning at all, as differences in prior knowledge may be influenced by differences in general intelligence. The “ability to understand complex ideas, to adapt effectively to the environment, to learn from experience, to engage in various forms of reasoning, to overcome obstacles by taking thought” (Neisser et al. 1996, p. 77) shows strong relationships with academic performance (Deary et al. 2007). In accordance with Cattell’s investment theory of intelligence (Cattell 1963, 1987), the higher people score on intelligence tests, the more likely they are to exploit learning opportunities and, thereby, accumulate knowledge to be used for future problem solving. This may affect all kinds of knowledge but likely to different degrees. If existing knowledge can be used in a new situation—e.g., by fact retrieval or by applying a well-established procedure—intelligence differences may play a minor role. If, however, conceptual knowledge has to be modified and/or restructured in order to be usable for further learning, intelligence differences likely come into play. In our study, the concept of proportionality was introduced, and learners had to use and adapt their mathematical knowledge about multiplication and division. 

The concept of intelligence was introduced in psychology to explain individual differences in learning outcomes despite equal opportunities, especially in content areas that emerged as a result of cultural development. This concerns verbal, numerical, and scientific literacy (Deary et al. 2007; Stern 2009), as well as skill at strategic games such as chess (Vaci et al. 2019). The latter longitudinal analysis controlled for the amount of exercise and showed a long-term influence of intelligence on chess performance, suggesting that more intelligent players can expand their lead through practice. Similar results were revealed in studies with highly knowledgeable learners scoring at the high end of the scale for general cognitive abilities (Makel et al. 2016; Berkowitz and Stern 2018). In these highly selective samples, existing differences in cognitive ability accounted for academic achievement and success. The advantage of intelligence for learning is twofold; intelligence facilitates the acquisition of knowledge, which, in turn, facilitates future learning. In this case, a rich-get-richer (or Matthew) effect is likely to occur. The gap between more and less intelligent learners likely increases over time (Witherby and Carpenter 2021) because intelligent learners may better exploit learning environments attended by all, and they may actively search for additional learning opportunities that help them to progress further. In our study, we attempted to disentangle the effects of these two settings by offering an early learning opportunity only to a portion of the learners. 

The onset of institutionalized education in schools strengthened the mutual relationship between intelligence and learning. As put by Snow (1989, p. 496): “Educational psychology now recognizes intelligence as education’s most important product, as well as its most important raw material”. Systematic instruction on reading, writing, and arithmetic and general cognitive capabilities support each other (Brod et al. 2017). In parallel, during the first years of school, individual differences in intelligence scores stabilize (Schneider et al. 2014), and these differences also affect the acquisition of more domain-specific yet broadly applicable competencies, such as scientific or proportional reasoning, which take years to develop. While achievement in these competencies is closely related to intelligence (Deary et al. 2007), some of the individual differences can also be traced back to differences in access to learning opportunities. This applies to both the effects of direct teaching (Staub and Stern 2002) and the indirect effects of preparatory activities in which children are given the opportunity to acquire prior knowledge that they can build on when developing more complex reasoning strategies.

Long-term effects of earlier preparatory activities have been shown for literacy (Schneider 2009) and mathematics (Hannula and Lehtinen 2005). For scientific reasoning, Schalk et al. (2019) revealed the broader transfer effects of physics teaching units on scientific reasoning in terms of the application of a control-of-variables strategy in the Swiss MINT Study. Transfer effects of early physics curricula were revealed by applying a test some months after the teaching units. In the current study, however, we went a step further by applying the preparation for future learning paradigm (Bransford and Schwartz 1999). By offering training on proportional reasoning using the example of density, we wanted to find out whether the group of students who had formerly undergone a basic curriculum unit on floating and sinking would be advantaged when learning about proportional reasoning. With our quasi-experimental field study, we attempted to disentangle the effects of intelligence from prior knowledge by controlling access to learning opportunities. Half of the participants had the opportunity to acquire conceptual knowledge in physics while the other half did not. How these earlier differences in learning opportunities affected later learning and how intelligence interacted with access to learning opportunities were investigated. 

### 1.2. Prior Knowledge as Mediator of the Relationship between Intelligence and Learning Outcomes

The positive correlations between intelligence, prior knowledge, and learning outcomes require further examination since there may be mediation effects. In general, running mediation analyses is justified when the direction of the causal impact is clear. This is the case for prior knowledge and intelligence, as the latter is likely to influence the acquisition of prior knowledge but not vice versa. The more intelligent people are, the more likely they are to acquire usable knowledge, which, in some cases, fully explains learning outcomes. In the case of a full mediation, intelligence only has this indirect effect. In case of a partial mediation, learning outcomes are markedly traced back to the investment of intelligence into prior knowledge, but there is an added value of intelligence beyond prior knowledge. This means that more intelligent learners can activate resources that go beyond the application of existing knowledge. This is likely the case if the goal of learning is the enrichment of existing knowledge. Reasoning ability must be deployed to recognize similarities between problems of similar abstract structure but with different superficial features. This was likely to happen in our study, as learners had to adapt their mathematical knowledge, as well as their conceptual knowledge, to the context (speed or density) used in the training on proportionality. In this case, a partial mediation of the relation between intelligence and learning outcome by prior knowledge was most likely. 

Depending on a student’s learning history, intelligence and prior knowledge may also interact with learning opportunities. More intelligent learners may use the necessary prior knowledge more efficiently and, thereby, gain from learning opportunities, whereas less intelligent students may not. On the other hand, learners with high prior knowledge may compensate for differences in intelligence. Such results were found in the seminal study from Schneider et al. (1989), who compared the impact of intelligence and prior knowledge on learning from a text on soccer. This content area allowed such questions to be properly addressed because people interested in soccer come from the entire range of intelligence. As soccer is based on complex but not very abstract rules, different from academic content areas, finding knowledgeable people at the lower end of the intelligence scale was easily possible. 

From what has been discussed so far, intelligence and prior knowledge are mutually dependent, and both contribute to learning outcomes in manifold ways. One can easily come up with plausible hypotheses, but it makes no sense to test them against each other without taking into account the characteristics of the content area and the sample. There will rarely ever be a general answer to questions like “What is more important for learning outcome, intelligence or prior knowledge?” (see also Simonsmeier et al. 2021) or “Does prior knowledge mediate or moderate the relationship between intelligence and learning outcome?” However, addressing such questions under more specific conditions can be very useful for better understanding individual differences in learning outcomes. These specifications may concern numerous aspects, among them, the type of knowledge to be acquired (conceptual, procedural, factual), the content area (e.g., STEM, language, humanities), characteristics of the sample (e.g., age, cultural, educational, or social background), or the kind of learning opportunity (classroom, informal, casual, etc.). In our study, we focused on a formal and transferable understanding of proportional reasoning among children at the end of elementary school. At this age, intelligence differences have largely stabilized, while proportional reasoning is still under development and likely responds to a targeted training. How intelligence, prior knowledge in mathematics, and specific knowledge about the physics concept of density affect learning outcomes was investigated. 

### 1.3. Proportional Reasoning: The Interaction between Formal Principles and Content-Specific Examples 

Understanding proportionality is not only important in mathematics but also in chemistry, biology, physics, economics, and many other disciplines (Fernández-Verdú and Llinares 2009). In mathematical terms, proportional reasoning involves comparing ratios within or between quantities based on the formula *a/b* = *c/d*, and this relationship is what we mean when we refer to proportionality in this article. It is necessary to understand that the relationship between the quantities is a multiplicative one and refers to a covariation. This implies that students learn that increasing *a* by a certain factor requires either multiplying *c* or *b* with the same factor or dividing *d* by this factor to establish the same ratio. Many students, however, erroneously compute differences rather than ratios (Inhelder and Piaget 1958; Tourniaire and Pulos 1985). Understanding proportions is the key to mastering fractions, decimals, and ratios, which are central topics of early secondary school mathematics classes. In the same vein, elementary school students’ competencies in solving proportional problems are highly predictive for secondary school performance (Siegler et al. 2012; Stern 2009).

Children already have knowledge about some proportional concepts such as speed or price per piece. However, many secondary school students still have difficulties in applying proportionality correctly as they cannot distinguish proportional from non-proportional situations (Boyer et al. 2008; Knox 2017; Van Dooren et al. 2010). According to the National Council of Teachers of Mathematics (2000, p. 217), “… facility with proportionality involves much more than setting two equal and solving for the missing term. It involves recognizing quantities that are related proportionally and using numbers, tables, graphs and equations to think about the quantities and their relationships”. 

One instructional method to facilitate students’ understanding of proportional reasoning is concreteness fading (Knox 2017; Kokkonen and Schalk 2020). This instructional method includes three phases. During the concrete phase, learners are faced with a familiar situation based on a proportional concept (e.g., a car travels 330 km in a 3 h trip). During the pictorial phase, learners are supported in using visual-spatial representations, such as diagrams, for depicting the mathematical structure of the situation dealt with during the concrete phase (e.g., drawing a time–distance diagram for motion assuming constant speed). In the abstract or idealized phase, learners are encouraged to transform their pictorial representation into a numerical equation and to apply the numerical relation to other concepts (e.g., dividing distance by time to calculate the speed).

The method of concreteness fading is an intuitively plausible way of teaching formal principles, and it has a long tradition in classroom practice (Kokkonen and Schalk 2020). It works when learners bring in the necessary prior knowledge that they can merge into a new competence under the guidance of the teacher, who has to take care of the activation of the relevant prior knowledge in an appropriate order. This means that the selection and the sequencing of the learning material is of particular importance in that respect. The first decision to be made concerns the concept and the situation to start with in the concrete phase. These have to be familiar to the students, but, at the same time, the learners should be able to detach themselves from the situational context when undergoing the pictorial and the abstract phase. Otherwise, they may activate personal experience that distracts from the actual learning goal because students might only remember the specific examples in which the knowledge remains situated. In such cases, students are not able to transfer their understanding from one example to another or more broadly to the abstract, general concept. 

A contextualized concept frequently used in the training of proportional reasoning is speed (e.g., Boudreaux et al. 2020; Boulanger 1976; Lamon 1993; Heller et al. 1989; Thompson 1994; Thompson and Thompson 1994; Wright 2011). Young children already have experience with fast and slow movements from using their toy vehicles, running contests, and many more situations. They might also know that increasing the speed means that one can cover a longer distance in a certain amount of time or that one arrives earlier at a particular destination. In late elementary school, students are also familiarized with measuring the quantities that form speed, which are time and distance. Whereas there are many good arguments to explain proportions using the context of speed, to the best of our knowledge, its effectiveness has never been contrasted by training using another context. 

In our study, we wanted to find out whether students who underwent a basic curriculum unit on the floating and sinking of objects in water and, thereby, learned about the concept of density, have an advantage if this concept is used as the context in training about proportionality. The concept of density resembles the concept of speed in core aspects, as both are based on continuous quantities that have a meaning in themselves (distance and time respectively volume and mass). However, different from speed, density rarely plays a role in children’s life. For their interaction with the physical world, it is more important whether an object is heavy and, therefore, cannot be removed or carried or whether it is light and, therefore, can be replaced (Smith et al. 1985). Many studies showed that, without systematic instruction, students rarely develop an understanding of density as the relationship between volume and mass before early adolescence (Hashweh 2016; Kohn 1993; Smith et al. 1985, 1992). However, undergoing a targeted instruction on density leads to a strong increase in conceptual understanding among elementary school students even at the age of eight (Hardy et al. 2006; Leuchter et al. 2014).

We wanted to find out whether the knowledge acquired in such learning environments is suitable for transfer to proportional reasoning. Support for expecting such effects comes from the seminal work of Barnett and Ceci (2002) on the likelihood of achieving transfer effects. The likelihood of transfer depends on the extent to which the learning and the transfer situation resemble each other in the dimensions of knowledge domain and the physical, temporal, functional, and social contexts, as well as the modality of testing. Whether transfer of knowledge can be expected or not has to be interpreted in the light of the learners’ prior knowledge (e.g., DiSessa 2006; Orrill and Brown 2012). Prior knowledge can enable efficient processing of incoming information in many ways. When incoming information meets existing knowledge, it is more likely to be remembered in the long run (Gobet and Simon 1998) and to be considered for problem solving (Dixon and Brown 2012). In our case, this means that students who learned about density in a formal school context should have been more likely to take advantage of this prior knowledge when faced with proportions in the example of density in a similar context. For example, knowing that, of two cubes that have the same volume but different weights, the heavier cube floats at a lower level or sinks in contrast to the other cube, might implicitly prepare students to recognize the relationship between weight and volume and to form a proportional concept.

Thus, when students have received systematic instruction on density and can build on prior experience in an academic context, they should be better prepared to learn about proportionality taught in the context of density. In contrast, when students deal with speed in their daily life, they do not necessarily think about the relation between distance and time. Real-life examples may hamper mathematical reasoning (Mevarech and Stern 1997; Stern and Mevarech 1996). At the same time, the similarities in the physical context of learning increase the likelihood of transfer (Barnett and Ceci 2002). As students learned about density in the classroom context, they are likely to activate the concept in a similar context. 

Transfer effects might even be amplified by the fact that, in both situations, learning gains are assessed by multiple-choice tests. The likelihood of transfer is also increased as the learners not only get training for the target content (proportional reasoning) but also for the source content (floating and sinking). This gives learners time to familiarize themselves with the target content, activate their prior knowledge, and then tailor it to the new content (see Barnett and Ceci 2002). Based on these arguments, we wanted to find out whether conceptual overlapping between a basic curriculum unit on floating and sinking applied in lower elementary school and training on proportional reasoning applied in upper elementary school particularly benefits proportional reasoning skills in relation to preparation for future learning. To disentangle the potential effects of early physics education and the effects of using density as an example in the proportional reasoning training, we applied a 2 × 2 design, described in the next section. 

### 1.4. Design, Research Questions, and Hypotheses 

All participants were in fourth or fifth grade when they underwent the training on proportional reasoning, applied in their classroom by an external teacher. At this age, the development of proportional reasoning abilities is steadily increasing, but it is a component of the school curriculum only at the end of elementary school. To further specify and confine the potential impact of prior knowledge, two parallel training schemes on proportional reasoning were developed; in one, speed was used as the context, and, in the other, density was used. Classes were randomly assigned to one of these conditions. 

Some of the students had participated in the Swiss MINT Study at an earlier time during elementary school and had undergone a basic curriculum unit on floating and sinking of objects in water and, thereby, had acquired prior knowledge on the concept of density. The described variations allowed the application of a 2 × 2 design with the factors basic curriculum unit on floating and sinking (*F&S* vs. *noF&S*) and intervention context (*density* vs. *speed*), resulting in four groups:

- *F&S/density*: Participants had learned about density in the basic curriculum unit on floating and sinking earlier in elementary school, and proportional reasoning was introduced by using the context of density;
- *F&S/speed*: Participants had learned about density in the basic curriculum unit on floating and sinking earlier in elementary school, and proportional reasoning was introduced by using the context of speed;
- *noF&S/density*: Participants had not undergone a basic curriculum unit in elementary school, and proportional reasoning was introduced by using the context of density;
- *noF&S/speed*: Participants had not undergone a basic curriculum unit in elementary school, and proportional reasoning was introduced by using the context of speed.

In the test of proportional reasoning applied at the end of the training on this topic, we expected that the *F&S/density* group would outperform all other groups. Learners of this group were most likely to make use of their prior knowledge on density and, thereby, advance their conceptual understanding when receiving training. On the other hand, it was unlikely that students who had not had the opportunity to learn about density beforehand would benefit from learning proportions from the example of this concept. Students who learned about proportionality in the context of speed but could only build on informal knowledge acquired through everyday experience would be less well prepared to transfer knowledge to the concept of proportionality.

In addition to the mentioned between-group difference in the mean scores of proportional reasoning, we planned analyses on the impact of prior knowledge and intelligence on gaining from the training on proportional reasoning. By applying a measure of general intelligence, on the one hand, we expected to confirm the strong relationship between intelligence and achievement in proportional reasoning. On the other hand, depending on prior knowledge, intelligence may reveal its potential to different degrees in the training based on the example of density or speed. To allow detailed analyses, the impact of prior knowledge was investigated at two different levels. First, our design allowed the experimental control of conceptual knowledge about density, as only half of the students underwent the basic curriculum unit on floating and sinking. By presenting a test on conceptual understanding of density, we wanted to find out whether this group was superior to the group of students who did not undergo the unit. We describe below that access to the physics unit can be considered a random assignment. Second, we included a broad test battery of mathematical knowledge, as multiplication, division, and mathematical modeling are a precondition for proportional reasoning. Individual differences in mathematical knowledge were expected to have a strong impact on the extent to which students gained from the training on proportional reasoning. We conducted two consecutive studies that we report next.

## 2. Study 1

### 2.1. Participants

Participants were recruited on the basis of school class via the Swiss MINT Study, a large, longitudinal study with more than 15,000 participants who underwent early physics education in elementary school. Six classes had completed the aforementioned basic curriculum unit on floating and sinking prior in earlier school years, while six classes were part of the waiting group and had not yet started the teaching unit. In Switzerland, students are assigned to schools according to their place of residence, so it is not the students or their parents who choose a particular school. It is likely that teachers taking part in the current study were interested in improving physics education at school. This means that students from the waiting group (*noF&S*) were also taught by teachers who were interested in improving early physics education. We minimized the differences between the student samples by parallelizing the recruitment areas of classes at the “waiting group” stage and the “applying the teaching unit” stage (rural, agglomeration, city, and average socioeconomic status of the school district). We randomized the intervention context between classes with comparable recruitment areas, thus, ensuring that effects of schools/cities were equally distributed across both intervention conditions. 

Students who participated in the Swiss MINT Study were already approved for participation with informed consent by their parents. Students who did not participate in a former curriculum of the Swiss MINT Study were provided with a consent sheet and information material for their parents. Students for whom informed consent was not given still participated in all lessons and tests, but we deleted their data.

The 251 participants were students (49% female; age at training: M = 10.73 years, SD = 0.55) from 12 classrooms at the end of grade 4 or at the very beginning of grade 5, which means there were three classes in each condition. The participants fell in the following cells of the 2 × 2 design: *noF&S/density*, n = 73; *noF&S/speed*, n = 54; *F&S/density*, n = 54; *F&S/speed*, n = 70 (these numbers already consider the exclusion of the 13 students from the data who did not participate in the training).

### 2.2. The Implementation of Early Physics Teaching Units in the Swiss MINT Study

Within the Swiss MINT Study, elementary school teachers were trained in early physics curricula developed by a team of science education experts from the University of Münster (Lemmen et al. 2008; Möller and Jonen 2005; Möller et al. 2007, 2008). The inquiry-based curricula included four different basic physics topics: floating and sinking, air and atmospheric pressure, sound and spreading of sound, and stability of bridges. The curricula were tailored to develop students’ content-specific conceptual knowledge on these four topics (Möller and Jonen 2005). Every curriculum required students to engage in experiments to explore the physics concepts. Aside from this inquiry-based material, the curricula put a focus on instructional guidance and teacher-led classroom discussion. For each curriculum, teachers who participated in the Swiss MINT Study received half a day of training that introduced the learning material and the educational concepts. Each of the curricula encompassed 15 classroom lessons (see Nussbaumer et al. 2017; Schalk et al. 2019).

The floating and sinking basic curriculum unit introduces the concepts of water displacement and object density to answer the question “Why does a large ship of iron float in water?” (Hardy et al. 2006, p. 310). To achieve this, the basic curriculum unit presents a sequence of topics that allows discovery learning by hands-on experience (Hardy et al. 2006). This sequence starts with experiments on material type and everyday objects, proceeding to the principle of water displacement and to a generalized concept of buoyant force (i.e., the Archimedean principle). The teacher supports the learners by guided inquiry, scaffolding, and reflective questions. Usually, the Archimedean principle is only taught in secondary school, when knowledge on mass, volume, density, and buoyant force is already present and the connection between physical quantities and scientific principles can be drawn. However, as Hardy et al. (2006) showed, the principle can be learned in elementary school, when learners have the chance to revise their misconceptions and integrate their knowledge on the different parts of the concept. Hardy et al. (2006) also presented a conceptual test that measures students’ misconceptions, everyday conceptions, and scientific conceptions. They suggested a combined sum score that reflects students’ misconceptions and scientific answers—Integrated Conceptual Understanding (ICU)—which we applied in the present study.

Within the floating and sinking basic curriculum unit, students encountered many examples of implicit proportional relations. For example, they immersed cubes of different materials but of the same size or cubes of the same material but with different sizes into water to compare how material and size influence floating ability. Through this inquiry-based approach, students obtained an idea of the concept of density. Therefore, in this basic curriculum unit, elementary school students gained experience in the domain-general concept of proportions in addition to the content knowledge of floating and sinking. This kind of learning is supposed to happen incidentally and denotes an excellent opportunity to test how such learning opportunities facilitate future learning on proportional concepts in general. None of the teaching units of the Swiss MINT Study included formal instruction about proportional relations. 

### 2.3. The Training on Proportional Reasoning

In the present study, the training on proportional reasoning (both speed and density) consisted of three lessons (45 min each) with the learning goals that students (1) understand that proportionality refers to a multiplicative covariation of variables, (2) can solve proportional missing value problems, (3) can draw proportional relations in Cartesian coordinate systems, (4) can solve proportional problems in the context of speed, density, and in transfer contexts, and (5) can solve problems in numerical and text form. 

The lessons were based on the idea of concreteness fading (Goldstone and Son 2005; Kokkonen and Schalk 2020). In the speed group, students were faced with toys that traveled the same distance in different times or operated at different speed, whereas, in the density group, students were shown cubes of the same size but different material and, thus, different weights. The students were faced with tasks including different combinations of time/distance or mass/volume. Students were guided in focusing on the underlying mathematical structure of the problem. We asked students to provide multiple solutions and representation strategies and, therefore, promoted the use of external representations in explaining the concept and in calculating proportions. The material and the teacher explicitly compared and contrasted the different solutions and representations (see Ziegler and Stern 2014, 2016). Furthermore, we tried to implement self-explanations (Jitendra et al. 2009, on schema-based instruction), whereby the training included phases of direct instruction alternating with phases of working alone and in pairs. As problem presentation influences task difficulty and often determines whether a student can solve a problem (Boyer et al. 2008), our participants solved and received feedback on two different problem types: comparison problems and missing values problems (Van Dooren et al. 2005). The two intervention contexts of speed and density were tightly parallelized, and the same numerical values were used in both of them. 

At the beginning of the lesson, the teacher introduced the topic either with cubes of different material or of different size (density context) or with two toy trains and a conveyor belt with different velocities (speed context). The conveyor belt was introduced in order to provide a second everyday life example as fewer possible disturbing factors could be imagined than could be imagined with trains. The students were encouraged to actively formulate hypotheses and conceptions about the topic. For example, they were asked which toy train would be faster and what one would need to manipulate and observe in order to determine which of the two toy trains was faster. The training material consisted of paper/pencil task sheets starting with general, related tasks such as comparing the velocities of real-world objects or the floating properties of certain objects. The aim of the material was to put focus on reinforcing multiplicative instead of additive processing of the relationships. Thus, different representations were introduced: descriptions of mathematical relationships in words, tables of values, graphical representations (e.g., Cartesian coordinate systems in which students had to draw proportional relationships; for a similar procedure, see Lehrer et al. 2001), and mathematical formulas. All materials aimed at making it clear that a ratio between two numbers is central for the concept of proportionality. Furthermore, the intervention contexts of density or speed were specified with definitional terms and mathematical relations (including fractional representations). The students solved various tasks, received in-between summaries, and always had the opportunity to ask questions.

### 2.4. Measures

**Figure 1:**
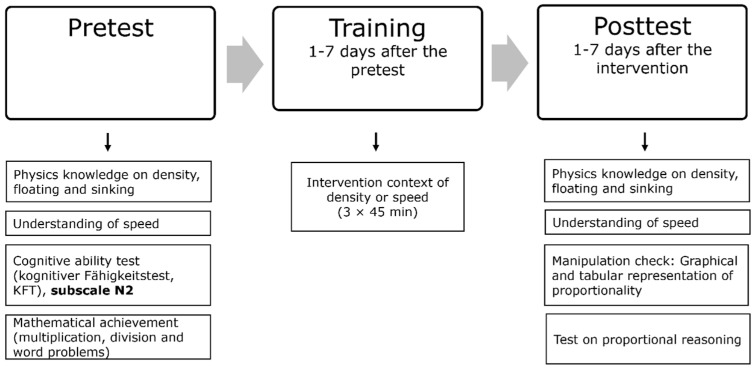
Schedule and overview of tests and the training.

**Figure A1:**
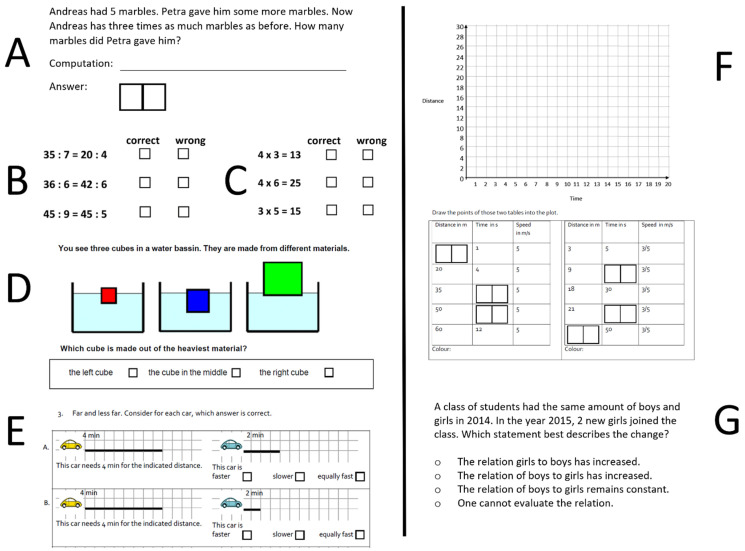
Example items of the measures in the pretest and posttest. (**A**) mathematical word problem; (**B**) division problems; (**C**) multiplication problems; (**D**) concept test on density; (**E**) concept test on speed; (**F**) manipulation check: table and Cartesian coordinate system for a proportional relation in the context of speed; (**G**) advanced proportional reasoning test. *Note*: The test material is available on https://osf.io/9tz3c/ (except the intelligence measure due to copyright reasons).

Knowledge was assessed with a pretest and a posttest, 1–7 days before and after the training. Figure 1 presents the time schedule of the proportionality training and the measures. See Figure A1 in Appendix A for example items for all measures.

#### 2.4.1. Measure for Manipulation Check

To check how much knowledge from the training on proportional reasoning was retained, we included a manipulation check in the posttest. This included drawing points on a Cartesian coordinate system (Felbrich 2005) to depict a proportional relation, which was part of the training. It also included tasks on calculating proportional relations separately for both intervention contexts, that is, speed and density. For each intervention context, the time limit was 8 min.

#### 2.4.2. Advanced Proportional Reasoning Test for Study 1

The dependent variable was the achievement in a test on proportional reasoning which was presented after the training. The test consisted of 25 multiple-choice items with four answer options. An example item was “In a box of chocolates, for 4 red chocolates there is one green chocolate. What is the ratio of green and red chocolates? (A) 4:1, (B) 1:4; (C) 4:5, (D) 1:5”.

#### 2.4.3. Concept Tests on Speed and Density 

We assessed knowledge about the concepts used as examples in the training on proportional reasoning in a test on understanding speed and density. For the concept test on density, we used a test developed by Hardy et al. (2006) on the topic of floating and sinking. The test resulted in a general score of Integrated Conceptual Understanding (ICU), which is calculated through misconceptions, which are scientifically incorrect, everyday conceptions, which are partially correct, and scientific conceptions, which are correct (see Hardy et al. 2006 for an extensive review). Students had 5 min to complete this test. 

For the concept test on speed, all students were presented with three tasks: In the first two tasks, they had to draw the distance which a car covers compared to another car in a given time. The proportions used were 1:3, 1:2, 1:0.5, and 1:0.25 for the first task and 3:2, 1:1.5, 1:3.5, and 3:1 for the second task. In the third task, they had to decide between two given distances and to indicate which car out of two was faster in eight items. Students had 5 min to complete this test. 

The tests were presented to both groups before and after the proportional reasoning training. Thus, the pretest allowed us to find out whether the students who had undergone early physics education had retained their knowledge on density. In addition, the posttest allowed us to find out whether the students gained from the training with regard to their conceptual knowledge.

#### 2.4.4. Tests on Prior Mathematical Achievement 

We assessed mathematical achievement through word problems, multiplication items, and division items. The word problems were ten open-answer tasks with one- and two-digit numbers using multiplicative and additive problems with a time limit of 10 min. They assessed mathematical modeling. The multiplication and division tasks were multiple-choice tasks involving the multiplication tables up to ten. The multiplication tasks were two times 22 items, with a time limit of 60 s for each part; the division tasks were 22 items with a time limit of 90 s. The students were instructed to answer as fast as possible and as accurately.

#### 2.4.5. Intelligence Test 

We used the Cognitive Ability Test (“Kognitiver Fähigkeitstest”, KFT, Heller and Perleth 2000), which is composed of subscales (numerical, verbal, and figural). Due to time constraints, we did not apply all subscales of this test but only the non-verbal figural analogies subscale N2 (Form A for fifth grade, total norms, 8 min), as this subscale showed the highest factor loadings on the *g*-factor of the whole test in the validation sample across all measurement points.

#### 2.4.6. Analysis

Data were analyzed with R (version 4.0.3, R Core Team 2020) using the RStudio environment (version 1.4.1717, RStudio Team 2021). We used the following packages (in alphabetical order): afex (Singmann et al. 2015), dplyr (Wickham et al. 2015), effsize (Torchiano 2017), emmeans (Lenth et al. 2018), equivalencetests (Ng et al. 2013), ggplot2 (Wickham 2016), modelsummary (Arel-Bundock 2021), tidyr (Wickham 2017), TOSTER (Lakens 2017a).

### 2.5. Results 

Means and standard deviations for all measures per group are presented in Table 1. The manipulation checks for density and speed showed that the vast majority of students learned what they were taught; in all groups, 62–67% of the students solved all problems correctly. Most measures showed low skewness (all below 1) and kurtosis (all below 2), and, except for some subgroups, the degree of violating the univariate normality assumption can be considered low. As expected, the *F&S* groups and the *noF&S* groups differed in concept knowledge about density in the pretest score (*F*(1, 245) = 56.25, *p* < .0001, η_G_^2^ = .19) and in the posttest score (*F*(1, 245) = 63.36, *p* < .001, η_G_^2^ = .21). This shows that learners who underwent the basic curriculum unit on floating and sinking had a sustained advantage in conceptual knowledge on density over students who did not. Our experimental variation of prior knowledge was effective. 

Across all groups there was an increase in the solution rate from the pre- to posttest on conceptual understanding of speed and density (solution rates increased by between .04 and .12 across groups, with both *p*-values < .001). The reasons for this are most likely repeated testing effects: the first application of the items may have stimulated spontaneous deeper thinking that slightly improved conceptual knowledge. 

**Figure 2:**
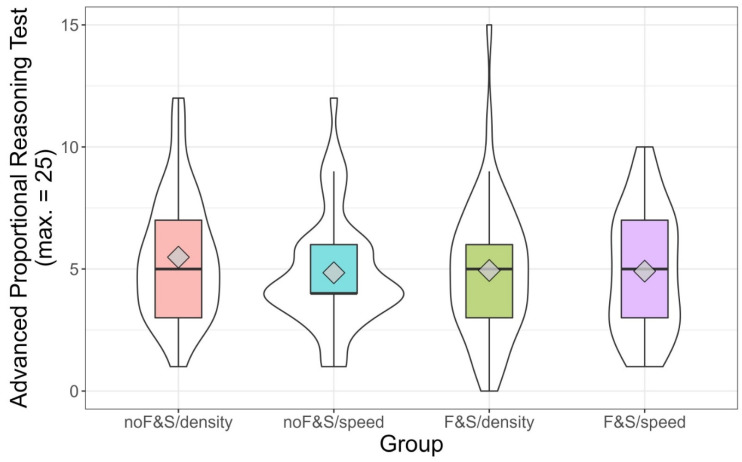
Score on the advanced proportional reasoning test by group in Study 1. Violin plots (white) and boxplots (colored). The diamond depicts the mean value per group.

The test on proportional reasoning appeared to be quite difficult, with mean solution rates of around .20. The maximum number of points that could be reached was 25, while the maximum found in our sample was 15 points. The score distribution on the proportional reasoning test presented after the training is depicted in Figure 2. Overall, no significant main effects were observed for the factors basic curriculum unit (*F*(1, 234) = 0.54, *p* = .46, η_G_^2^ = .002) and intervention context (*F*(1, 234) = 1.04, *p* = .31, η_G_^2^ = .004). Additionally, the interaction between these two factors was not significant (*F*(1, 234) = 0.87, *p* = .35, η_G_^2^ = .004). In order to check whether no meaningful difference existed between the groups, we calculated a Wellek’s F-test for equivalence (Wellek 2010) using η^2^ of 0.01 as the smallest meaningful effect. This equivalence test was also not significant (*F*(3, 247) = 0.04, *p* = .56), yielding inconclusive evidence about the group effect.

Thus, achievement differences in proportional reasoning after the training were neither explained by the context used during the training (speed or density) nor by having undergone a basic curriculum unit nor, as expected, by the combination of both. 

**Figure 3:**
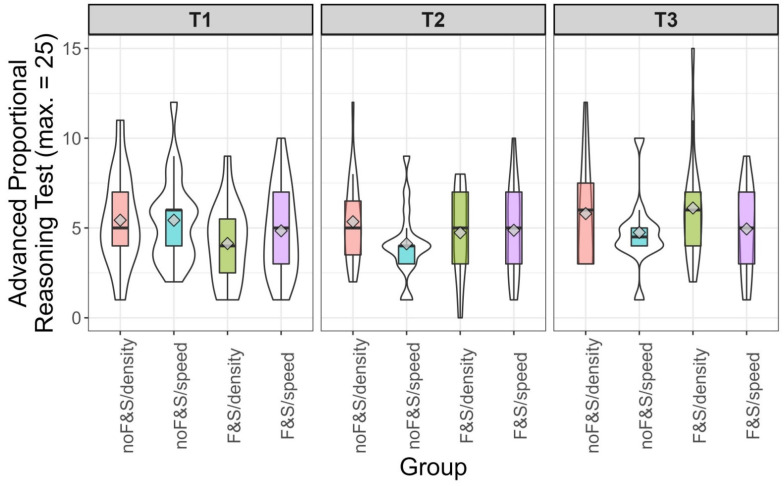
Score on the advanced proportional reasoning test by intelligence tercile and group in Study 1. Violin plots (white) and boxplots (colored). The diamond depicts the mean value per group.

The correlation between intelligence and achievement in the advanced proportional reasoning tests varied between the groups and was highest in the *F&S/density* group (*r* = .37, *p* = .01). In the other three groups, it varied between *r* = −.14 and *r* = .08. To further scrutinize potential interactions between intelligence and group, we split intelligence scores into terciles. Figure 3 depicts the means, medians, and the distribution of the scores in the advanced proportional reasoning test. In the *F&S/density* group, the increase from the lowest to the highest tercile was most obvious. In the upper third of intelligence, some students of this group reached the highest scores in proportional reasoning. 

As in general, the group differences were rather negligible, and, for reasons of statistical power, we report the correlations for the entire sample (in Table 2).

### 2.6. Discussion of Study 1

We investigated whether students who participated in a basic curriculum unit on floating and sinking and, thereby, learned about the concept of density could profit more from their prior knowledge in training on proportionality than students who had not undergone the basic curriculum unit. The manipulation checks showed that students were able to calculate proportional relations and depict them in Cartesian coordinate systems in both intervention contexts, speed and density. However, a more general transfer of proportionality was difficult for the students. On the other hand, the analyses did not indicate equivalence of the effects between the groups; a result that encouraged us to run further analyses. Descriptive analyses suggested that there might be interactions between intelligence and making use of learning opportunities. Learners scoring in the upper third of intelligence could realize their potential best if assigned to the *F&S/density* group. Although the statistical power was not strong enough for thorough conclusions, and the results were different from our expectations, several findings encouraged us to apply the same design to a larger sample from the Swiss MINT Study. In particular, we were encouraged by the between-group differences in conceptual understanding of density. Students who had undergone early physics education retained their knowledge about density after years, which means that the preconditions for our design were fulfilled. In Study 2, we eliminated the following weaknesses of Study 1: First, the test on advanced proportional reasoning, which was the major outcome variable, was too difficult and, therefore, probably did not discriminate sufficiently between learners. Second, the teacher (second author) was involved in planning the study and was aware of the hypotheses, which may have caused an unconscious bias. Third, not enough classes, and, thereby, participants, were included to ensure the statistical power necessary for mediation analyses at group level.

## 3. Study 2

Prior to data collection, we preregistered Study 2 with hypotheses, design, and methods with the Open Science Framework (https://osf.io/ruyf8/). The hypotheses were based on the hypotheses from Study 1, but, also, new hypotheses were formulated.

We expected the basic curriculum unit on floating and sinking to have a significant main effect on the proportional reasoning test. Moreover, we expected a significant interaction between the basic curriculum unit and the intervention context. We predicted a particular advantage for the more familiar intervention context of density for those who underwent the basic curriculum unit on floating and sinking. Therefore, we hypothesized that the group who participated in the basic curriculum unit and had density as intervention context (*F&S/density*) would outperform the other groups on domain-general knowledge about proportionality. We had no specific hypotheses concerning the differences between the other three groups. We, furthermore, registered our expectations regarding the manipulation checks on the items dealing with the specific topic of speed and density. We expected that the group with density as an intervention context would perform better in the density items, and the group with speed would perform better in the speed items.

In addition to the between-group comparison of mean achievement, the statistical power of Study 2 also allowed us to run mediation analyses. We, therefore, investigated, for each of the four groups, whether the relation between intelligence and achievement on the advanced proportional reasoning test presented after the training was fully or only partly mediated by prior knowledge. We presented students with a test on mathematical knowledge, including multiplication, division, and word problems—tasks considered an important basis for proportional reasoning no matter which example was used in the training. We expected similar mediation effects for the four groups, as they all underwent comparable mathematics instruction. The second measure of prior knowledge that we included was a test on the conceptual understanding of density with which students entered the training. A substantial relation between intelligence and the pretest score was expected for only the two *F&S* groups, as only these students were given the chance to acquire conceptual knowledge about density. In accordance with the investment theory of intelligence, the higher people’s intelligence, the more they are expected to gain from learning opportunities. However, only the *F&S/density* group could make use of their knowledge about density during the proportional training. In this group, knowledge about density would, therefore, likely mediate the relationship between intelligence and achievement in the advanced proportional reasoning test. 

### 3.1. Method 

#### 3.1.1. Measures

The measures were the same as in Study 1, with the exception of the advanced proportional reasoning test. To approach the problem of the test of proportional reasoning being too difficult in Study 1, we developed new test items. We piloted these new items with one class of sixth graders (n = 18, 12–12.8 years old). We presented them with 28 items in two different orders for which they were given enough time to answer all items (40 min). After selecting those items with a high discriminatory power and solution rates between 0.10 and 0.90, we ended up with a final test of 17 multiple-choice items with 4–5 answer options. An example item was: “Michael buys 18 peaches. If 5 peaches cost CHF 4, how much money does he need? (A) CHF 11.6; (B) CHF 14.4; (C) CHF 16.5; (D) CHF 22.5”. 

#### 3.1.2. Participants and Procedure

We aimed at a sample size that was larger than the sample from Study 1. As most of teachers of classes involved in the Swiss MINT Study were quite interested in the study, the sample resulted in 580 students from 29 fifth-grade classes from the north and east of Switzerland. We excluded 14 students from the data, as they did not participate in the training. In line with our preregistration, these were the only cases which we excluded (aside from those with no consent for participation). The mean age of the students was 11.24 years (SD = 0.49) at the time of the training, and 54% were female.

Fifteen classes were randomly assigned to the context of density, and 14 classes were instructed with the context of speed. The participants fell in the following cells of the 2 × 2 design: *noF&S/density*, n = 141; *noF&S/speed*, n = 165; *F&S/density*, n = 146; *F&S/speed*, n = 114. Each class was paid CHF 150 for participation, and we provided the teachers with the lesson material for their own use. A teacher with 34 years of teaching experience in elementary and secondary schools in Switzerland instructed all participating classes in the unit on proportional reasoning. This teacher had no prior association with the authors, and she was not aware of the research questions and hypotheses. She was trained by the second author, who taught the classes in Study 1. Trained research assistants who were not aware of the hypotheses conducted the pre- and posttests.

### 3.2. Results

We first present the descriptive statistics and the analyses of the group differences in mean performance. In a second step, we present mediation analyses to better understand how intelligence and prior knowledge contribute to gaining from the training on proportional reasoning. 

#### 3.2.1. Mean Performance of the Groups

The manipulation check revealed that a considerable number of students had learned what they were taught. The students were good at the density task, in which 64% of the students solved all problems correctly but less so for the speed problems, in which only 40% of the students solved all problems correctly. However, solution rates over 0.50 clearly showed that the instruction was effective (Table 3). In our preregistration, we expected students to be best in the context in which they were instructed. However, the density groups did not perform significantly better in the density manipulation check (*t*(529.87) = 0.33, *p* = 0.74). The speed group did show a significant advantage in the speed manipulation check (*t*(527.43) = 2.88, *p* = 0.004). Despite the deviation from our expectations regarding the density manipulation check, most children learned what they were taught. 

Table 3 depicts the mean solution rates and internal consistency of our measures by group and over all groups. Most measures showed low skewness (all below 1) and kurtosis (all below 2) and, except for some subgroups, the degree of violating the univariate normality assumption can be considered low. The internal consistency of all scales was acceptable to high. The revised test on proportional reasoning had an acceptable-to-good internal consistency (ω_t_ = 0.77). The solution rates of most scales were in a good range of 0.40 to 0.60, whereas the knowledge of density depicted a floor effect with low solution rates for those groups without the basic curriculum unit. 

In our preregistration, we expected the groups to not differ substantially in the covariates of intelligence and mathematical competencies. This was tested with equivalence tests (Lakens 2017b), in which we considered differences lower than a Cohen’s *d* of .15 as negligible. However, none of the equivalence tests was significant (all *p*-values > .09) and, thus, did not demonstrate equivalence of the groups concerning these covariates. The a-priori-defined cut-off of *d* = .15 was probably too conservative. 

**Figure 4:**
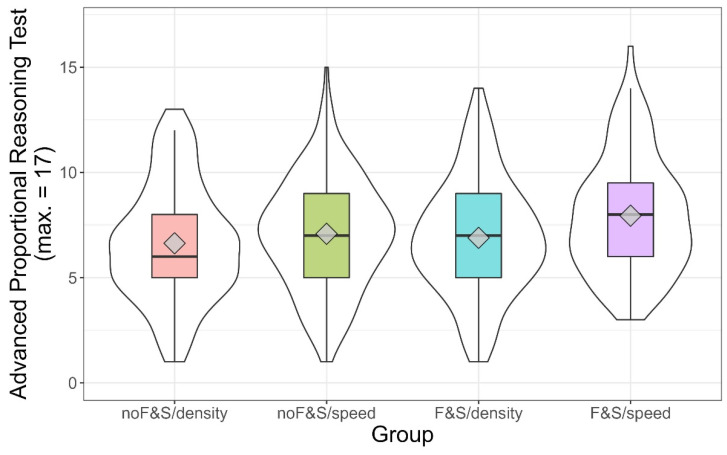
Score in proportional reasoning test by group in Study 2. Violin plots (white) and boxplots (colored). The diamond depicts the mean value per group.

To find out whether the curriculum on floating and sinking was sustainable, we compared the *F&S* groups against the *noF&S* groups, showing a clear advantage (*t*(485.24) = 13.14, *p* < 0.001, *d* = 1.15, 95% CI (0.96, 1.33)). Similar to Study 1, we observed a small increase in conceptual understanding of speed and density (solution rates increased by between .00 and .12 across groups, with both *p*-values < .01). The means and distributions of the advanced proportional reasoning test for the four groups are depicted in Figure 4. Contrary to Study 1, there was a main effect for intervention context (*F*(1, 534) = 10.12, *p* < .01, η_G_^2^ = .019), as well as for the basic curriculum unit (*F*(1, 534) = 5.59, *p* = .02, η_G_^2^ = .01). The interaction between intervention context and the basic curriculum unit was not significant (*F*(1, 534) = 1.62, *p* = .20, η_G_^2^ = .003). We found that training in the context of speed produced better outcomes than using density as the context. Moreover, children who had undergone early physics education performed better than those who had not. Contrary to our expectations, it was the *F&S/speed* group that outperformed all other groups (contrast: *t*(534) = 3.66, *p* < .001), while the *F&S/density* group did not outperform any other group (contrast: *t*(534) = −1.22, *p* = .22). 

**Figure 5:**
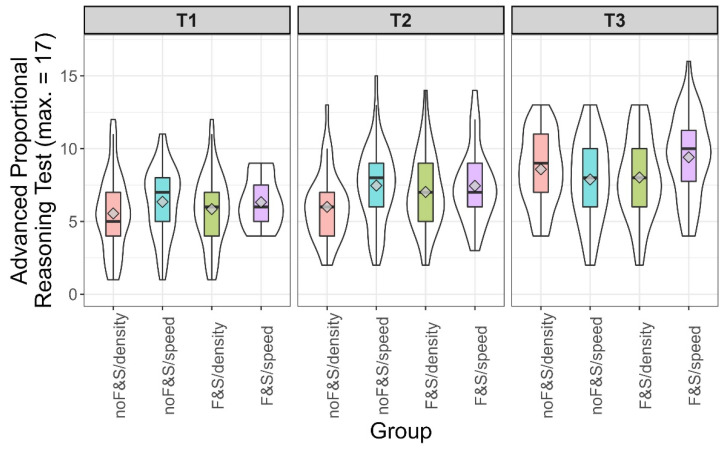
Score on the advanced proportional reasoning test by intelligence tercile and group in Study 2. Violin plots (white) and boxplots (colored). The diamond depicts the mean value per group.

As some of the results differed from our preregistered expectations, we, again, ran post hoc analyses to find out whether intelligence affected the extent to which learners gained from different learning opportunities. Figure 5 shows descriptive information on the achievement in the four groups split into terciles of intelligence. At a glance, it is obvious that the expectation raised in Study 1, in which the *F&S/density* condition was particularly beneficial for the learners scoring in the upper third of the intelligence scale, could not be confirmed in Study 2. In the range of medium intelligence, the students who underwent the basic curriculum unit and received the training in the context of density were advantaged over those who did not undergo the basic curriculum unit (one-sided *t*-test *t*(75.09) = 1.84, *p* = 0.03, *d* = 0.42). Yet, we are aware of the post hoc character of this analysis. Whereas the overall picture in Figure 5 does not deliver a consistent picture, it is obvious that teaching proportionality in the context of density in classes which did not undergo the basic curriculum unit (*noF&S/density*) was disadvantageous for students, except for those scoring in the upper third of the intelligence scale. 

#### 3.2.2. Correlations and Mediation Analysis

The correlations for the variables are reported in Table 4 by group. All correlations were positive, and, except for some relations of the concept of density with intelligence and mathematical achievement in some subgroups, they all reached significance.

**Figure 6:**
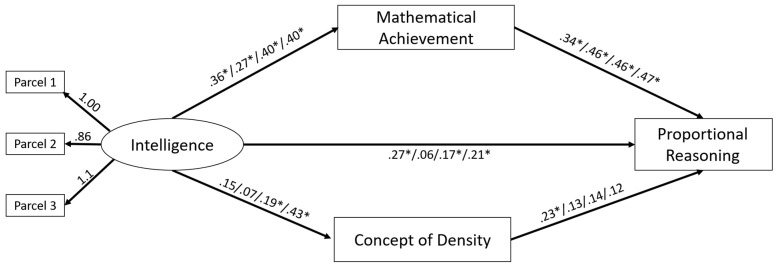
Mediation model for Study 2. Intelligence is modeled as a latent construct reflected in three item parcels. Values on the left arrows show factor loadings on these parcels. The other values depict standardized estimates per group separated by forward slashes (*noF&S/density*; *noF&S/speed*; *F&S/density*; *F&S/speed*). Asterisks indicate significance at *p* < 0.05.

To address the mediating role of prior knowledge between intelligence and proportional reasoning, we tested the mediation model depicted in Figure 6. We modeled intelligence as a latent construct, eliminating measurement error (Borsboom 2008) as we regarded it as a reflective construct. To enhance model parsimony, we used an item-parceling approach with three parcels (Matsunaga 2008). We did not model the other variables latently, as we regarded knowledge as a formative construct that is assessed via the specific items (Stadler et al. 2021; Taber 2018). Instead, we aggregated the scores from the multiplication, division, and word problems into a mean score of mathematical achievement to compare mathematical prior knowledge to physics prior knowledge. In the mediation model, we included intelligence as (latent) predictor, mathematical achievement and conceptual knowledge in density as mediators, and proportional reasoning as a dependent variable. To fit the mediation models, we used the lavaan package (Rosseel 2012). To estimate the indirect effect, we used robust Monte Carlo confidence intervals via a function from the semTools package (Jorgensen et al. 2021). Other than that, we used the same statistical packages as in Study 1. The model showed a good fit for the data (robust RMSEA = .039; CFI = .995; SRMR = .037) and explained the following amounts of variance in the advanced proportional reasoning test: *noF&S/density*, 34%; *noF&S/speed*, 25%; *F&S/density*, 32%; *F&S/speed*, 39%. The total effects in all groups were significant.

As shown in Figure 6, the relation between intelligence and proportional reasoning was partly mediated by mathematical achievement in most groups. To estimate the confidence intervals of the indirect effects, we, again, used bootstrapping. The resulting estimates are depicted in Table 5. All indirect effects of mathematical achievement were found to be robust (i.e., the 95% CI did not include 0). That is, intelligence affected mathematical achievement, which, in turn, affected proportional reasoning. The direct effect of intelligence also showed robust influence in three groups. This indicated that the mediation through prior knowledge was partial, and intelligence explains additional variance beyond prior knowledge. Regarding the indirect effects of density knowledge, the estimates were rather small, and the confidence intervals did not exclude the possibility that such prior knowledge might have had small negative or positive effects on proportional reasoning.

### 3.3. Discussion of Study 2

In Study 2, we remedied the fact that Study 1 included an overly difficult test. We managed to develop a test with a mean solution rate of around 0.40, which reliably depicted differences in performance. We, again, investigated whether attending a basic curriculum unit on floating and sinking enabled students to better exploit training on proportional reasoning with density as an example. Results on the conceptual understanding of speed confirmed our assumption that this concept was familiar to our students, while the concept of density was only familiar to students who attended the early physics curricula. However, contrary to our hypotheses, we could not prove an overall benefit for proportional reasoning in the case of learners who had undergone early physics education and later learned proportionality in the context of density. Rather, the best performance was achieved by students who had undergone the basic curriculum unit on floating and sinking and learned proportions in the context of speed. A post hoc explanation could be that students of this group could make use of two proportional concepts, which conforms to previous results which suggested that presenting two different examples is particularly helpful for deriving formal principles (Alfieri et al. 2013; Gentner 2010; Schalk et al. 2016). As all participants were presented with a conceptual test on density before the training, this most likely activated prior knowledge among the *F&S* learners. Entering the training on proportional reasoning after having revisited the content of density may have encouraged some learners to draw connections between speed and density. As the advantage of the *F&S/speed* group was particularly obvious among students who scored in the upper third of the intelligence distribution, this confirmed our claim that higher intelligence facilitates knowledge transfer beyond directly perceivable similarities between content areas. We are, however, aware that our results are not strong enough to definitely confirm this claim. 

Introducing proportionality in the context of density for classes who had not learnt about this concept in advance was ineffective for the vast majority of learners. Only those scoring at the higher end of the intelligence scale could make use of this setting. Compared to the use of speed, the use of density was inferior under all conditions. 

The statistical power of Study 2 allowed us to run mediation analyses in order to find out the extent to which intelligence was mediated by prior mathematical achievement, as well as by prior knowledge on density. We found, for all but one group, that the correlation between intelligence and proportional reasoning was only partly mediated by prior knowledge, which suggests that intelligence has an impact on how efficiently prior knowledge is adapted to learning about proportional reasoning. It was only for the *noF&S/speed* group that the relation between intelligence and proportional reasoning was fully mediated by mathematical achievement. The relatively low correlation between intelligence and mathematical achievement suggests that the profile of this group differed in this aspect from the other three groups. We refrain from further interpretations of the results of this group. 

For the groups *noF&S/density*, *F&S/density*, and *F&S/speed*, the mediation via mathematical achievement was quite similar: the correlation between intelligence and proportional reasoning was only partly mediated by mathematical achievement, as a direct impact of intelligence remained. This result suggests that mathematical achievement is necessary for gaining from training on proportionality, but, when intelligence is higher, the learning opportunity can be used more effectively. 

The role of conceptual knowledge of density as a mediator between intelligence and proportional reasoning was expected to be different for the four groups: the students in the *noF&S/density* group received no early physics instruction that would have given them the opportunity to invest their intelligence in the subject. The students of this group were particularly challenged (also reflected in the relatively low mean), as they had to learn proportionality on the concept of density, which was unfamiliar to them. In this group, the impact of intelligence remained strongest among all groups, and it was the only group where it had a direct impact on conceptual understanding of density. Obviously, only students scoring highly on the intelligence task and who had some prior knowledge on density had the chance to excel. 

For the two groups who had undergone early physics education, the mediation analyses revealed unexpected results. In both groups, intelligence had an impact on students’ prior knowledge of density. This was plausible, as intelligence can be expected to influence the representation and the retrieval of knowledge acquired in the basic physics curriculum unit. However, conceptual knowledge of density did not mediate the relation between intelligence and proportional reasoning in the *F&S/density* group. That differences in prior knowledge on density did not directly impact achievement in proportionality even after having undergone the training might be due to threshold effects; in order to make use of the training, a certain amount of conceptual understanding is needed. However, its use depends on intelligence. 

## 4. General Discussion

In the two studies, we focused on learning opportunities that were supposed to promote proportional reasoning, a broader formal competence applicable to many content areas. Whereas proportional reasoning emerges as a consequence of general cognitive development, and achievement differences are related to intelligence (Deary et al. 2007; Stern 2009), formal instruction is supposed to give the finishing touch. The goal of our study was to research the interaction between intelligence and prior knowledge on the exploitation of a training on proportional reasoning. Prior knowledge in mathematics was expected to have a strong impact on learning. In addition, we varied the availability of conceptual knowledge about density, as well as its usability, during the training on proportional reasoning. In this training, proportionality was taught either in the context of speed or of density. We applied a 2 × 2 design with the factors *F&S* (whether or not students had undergone a basic curriculum unit on floating and sinking earlier in elementary school, in which the concept of density was introduced in a non-formal manner) and context used to introduce the formal concept of proportionality (density or speed). 

The students in this study were about 11 years old, an age when strategies for solving proportional problems are under development and may change rapidly (Christou and Philippou 2001; Fuson and Abrahamson 2005). Students were expected to have mathematical knowledge, as well as knowledge about situations that imply proportional concepts. Having opportunities to integrate these sources of knowledge was likely to promote proportional reasoning. 

Our design was special with regard to several aspects. Firstly, in three lessons, we gave all participants the chance to activate their prior knowledge and to further advance their conceptual understanding before being tested. Our main outcome measure was a test on proportional reasoning, which measured the application of the concept to new situations. The training was built on proven educational techniques such as concreteness-fading (Knox 2017), comparing and contrasting (Ziegler and Stern 2014, 2016), and self-explanations (Jitendra et al. 2009). However, it goes without saying that three lessons are not enough to teach proportional reasoning from the scratch. Especially from the perspective of mathematics education, the finding that many students were not able to transfer their knowledge about proportionality to new contexts was not surprising. This is in line with studies showing that proportional reasoning takes years to develop (Boyer et al. 2008; Christou and Philippou 2001; Vanluydt et al. 2020) and that transfer does not come cheap (e.g., Bransford and Schwartz 1999; Barnett and Ceci 2002). However, as we studied an age group that is known for undergoing major changes regarding proportional reasoning, even a three-lesson training scheme was likely to have had an effect. In particular, it was expected to activate students’ mathematical knowledge, as well as their knowledge about concepts, that can be used to make sense of the representations and ideas presented during the training. To put it differently, our design increased the likelihood that existing knowledge is activated and used. 

Secondly, we applied the preparation for future learning paradigm (Bransford and Schwartz 1999). By using data from the Swiss MINT Study, we controlled for prior knowledge, as only a random portion of the students had undergone a basic curriculum unit on floating and sinking and, thereby, had learned about the concept of density. We hypothesized that students who underwent the basic curriculum unit on floating and sinking and who were taught the concept of proportionality in the context of density (*F&S/density group*) would develop a deeper understanding of proportionality appropriate for transfer. Therefore, our design allowed us to disentangle intelligence and prior knowledge on density in an experimental setup. 

Both studies revealed that students who underwent the early physics curriculum unit retained their knowledge about the concept of density. At the same time, however, knowledge about density did not have the predicted boosting effect in the *F&S/density* group. Whereas Study 1 at least raised hope that students scoring in the upper level of the intelligence scale would particularly gain from this condition, the statistically sufficiently powered Study 2 revealed that teaching proportionality in the context of speed was superior compared to teaching it in the context of density. Contrary to our expectation, this was also the case for learners who had undergone the basic curriculum unit on floating and sinking. We could rule out that this was the case because learners had forgotten about density, as a test on understanding density revealed a better outcome for those who had undergone the basic curriculum unit compared to those who had not. Future research has to show whether the post hoc explanation presented in the discussion of Study 2 applies. 

Our data also showed that teaching proportionality in the context of density in classrooms that had not undergone the basic curriculum unit on floating and sinking was disadvantageous for those not scoring at the upper third of the intelligence scale. This result shows the importance of building on prior knowledge when using examples.

We further looked for the relation between intelligence, prior knowledge in mathematics, and conceptual knowledge of density with regard to their impact on proportional reason after training. As expected, we found mathematical knowledge had a strong impact on proportional reasoning. Proficiency in multiplication, division, and word problem solving form an important basis for proportional reasoning. In accordance with the literature (e.g., Deary et al. 2007; Kyttälä and Lehto 2008), these competences were substantially related to general intelligence. Contrary to our expectations, however, conceptual content knowledge on density did not affect proportional reasoning in the predicted way in the *F&S/density* group. Although both *F&S* groups retained knowledge about density, achievement differences in the test on understanding density did not affect achievement differences in proportional reasoning. 

The major goal of the mediation analyses was to find out whether differences in intelligence had an effect on learning beyond prior knowledge. We had hypothesized that this was the case, as mathematical knowledge and conceptual knowledge about density had to be extended and restructured in order to be used for proportional reasoning. A partial mediation was most plausible, as all the kinds of prior knowledge we included required extension and restructuring in order to be useful for proportional reasoning. This was confirmed; although mathematical knowledge was, in fact, a strong mediator of the relationship between intelligence and proportional reasoning in all groups, in all but one group, there was an independent contribution of intelligence. 

This conclusion, however, is subject to reservations, as our measures of prior knowledge were precursors of proportional reasoning (e.g., multiplication and division) rather than proportional reasoning itself. We cannot rule out that the impact of intelligence would have disappeared if we had included a measure of prior knowledge. That we did not administer a pretest on proportional reasoning before the training started can be considered a drawback of our study. Since our focus was on the interaction between intelligence and prior knowledge in a new learning context, we used the time available for tests on mathematical precursors and knowledge of speed and density. Using the same test on proportional reasoning before and after the training would have required a different approach, as our test included new forms of representation introduced during the training with which students were not expected to be familiar beforehand. Therefore, a pretest on proportional reasoning should consist of problems that can be solved without formal instruction. Finding out whether the contribution of intelligence prevails if such tests are included is left to future research. 

The concept of intelligence was invented in psychology more than 100 years ago to explain differences in learning outcomes found between students despite comparable learning opportunities provided at school. Not all of these differences could be explained by family background, as many children from the humblest backgrounds learned to read, write, and do arithmetic with ease. In the 1980s, the explanatory value of intelligence was called into question by results that showed the importance of prior knowledge for future learning (Walker 1987; Weinert et al. 1989). In the meantime, knowledge and intelligence were no longer used as opposing explanations for individual differences in learning outcomes (Lauermann et al. 2020; Vaci et al. 2019). Mediation models, such as those applied in our study, suggested that, instead of “intelligence versus prior knowledge”, the relation between both concepts is better understood as “intelligence via prior knowledge”, as proposed by Cattell’s investment theory (Cattell 1987). Our results, however, showed that, even for a formal competence like proportional reasoning, this is not the whole story. Mediation models showed that intelligence made a unique contribution to exploiting the training on proportional reasoning. Future research should focus on individual differences in the process of learning while undergoing training. 

## Figures and Tables

**Table 1:** Mean solution rate per group and for total sample in Study 1.

Measure	ω_t_	*noF&S*		*F&S*		Total
		Density	Speed	Density	Speed	
Advanced Proportional Reasoning Test	.60	0.22 (0.10)	0.19 (0.09)	0.20 (0.11)	0.20 (0.1)	0.20 (0.10)
Concept of Density_pre_	.81–.85	0.20 (0.20)	0.12 (0.13)	0.42 (0.23)	0.34 (0.27)	0.27 (0.24)
Concept of Density_post_	.80–.86	0.29 (0.17)	0.21 (0.14)	0.54 (0.20)	0.42 (0.26)	0.36 (0.23)
Concept of Speed_pre_	.70	0.42 (0.18)	0.43 (0.18)	0.49 (0.19)	0.44 (0.19)	0.44 (0.19)
Concept of Speed_post_	.66	0.50 (0.17)	0.51 (0.16)	0.53 (0.20)	0.55 (0.20)	0.52 (0.18)
Intelligence (T-values)	.96	47.82 (8.31)	46.9 (9.9)	50.13 (7.06)	49.49 (6.09)	48.58 (7.93)
Mathematical Achievement	.77–.93	0.52 (0.19)	0.39 (0.14)	0.49 (0.18)	0.43 (0.15)	0.46 (0.17)
Manipulation Check Density	-	0.92 (0.24)	0.74 (0.34)	0.74 (0.35)	0.80 (0.29)	0.81 (0.31)
Manipulation Check Speed	-	0.93 (0.21)	0.74 (0.33)	0.74 (0.40)	0.85 (0.27)	0.83 (0.31)

Note. Standard deviation in parentheses. ωt depicts McDonald’s/Revelle’s omega total of the scale, assuming one latent factor. As the manipulation check consisted of one item, no internal consistency is reported.

**Table 2:** Bivariate correlations in Study 1.

Variable	Intelligence	Mathematical Achievement	Concept of Density
Mathematical Achievement	.16 **		
Concept of Density	.18 **	.25 ***	
Advanced Proportional Reasoning Test	.07	.29 ***	.04

Note: * *p* < 0.05, ** *p* < 0.01, *** *p* < 0.001.

**Table 3:** Mean solution rates (SD) per group and for total sample in Study 2.

Measure	ω_t_	*noF&S*		*F&S*		Total
		Density	Speed	Density	Speed	
Advanced Proportional Reasoning Test	.77	0.39 (0.16)	0.42 (0.15)	0.41 (0.16)	0.47 (0.16)	0.42 (0.16)
Concept of Density_pre_	.84–.87	0.28 (0.19)	0.25 (0.16)	0.45 (0.20)	0.54 (0.24)	0.37 (0.23)
Concept of Density_post_	.79–.85	0.40 (0.18)	0.29 (0.15)	0.53 (0.20)	0.56 (0.23)	0.43 (0.22)
Concept of Speed_pre_	.73	0.57 (0.18)	0.58 (0.18)	0.56 (0.19)	0.59 (0.18)	0.58 (0.18)
Concept of Speed_post_	.67	0.57 (0.18)	0.59 (0.17)	0.60 (0.18)	0.64 (0.18)	0.60 (0.18)
Intelligence (T-values)	.97	50.87 (9.14)	49.82 (8.26)	49.82 (9.40)	52.29 (9.57)	50.59 (9.09)
Mathematical Achievement	.84–.93	0.44 (0.16)	0.45 (0.14)	0.44 (0.15)	0.49 (0.17)	0.45 (0.16)
Manipulation Check Density	-	0.78 (0.34)	0.79 (0.33)	0.83 (0.30)	0.85 (0.28)	0.81 (0.32)
Manipulation Check Speed	-	0.59 (0.39)	0.71 (0.33)	0.67 (0.36)	0.73 (0.33)	0.67 (0.36)

Note. Standard deviation in parentheses. ω_t_ depicts McDonald’s/Revelle’s omega total of the scale, assuming one latent factor.

**Table 4:** Bivariate correlations per group in Study 2.

Pairs of Variables Correlated	*noF&S/* *Density*	*noF&S/* *Speed*	*F&S/* *Density*	*F&S/* *Speed*
Intelligence/ Mathematical Achievement	.39 ***	.20 **	.34 ***	.44 ***
Intelligence/ Concept of Density at Pretest	.16 *	.03	.13	.39 ***
Intelligence/ Advanced Proportional Reasoning Test	.53 ***	.16 *	.36 ***	.48 ***
Mathematical Achievement/ Concept of Density at Pretest	.18 *	.05	.32 ***	.27 ***
Mathematical Achievement/ Advanced Proportional Reasoning Test	.49 ***	.48 ***	.55 ***	.57 ***
Concept of Density at Pretest/ Advanced Proportional Reasoning Test	.33 ***	.16 *	.24 ***	.31 ***

Note. * *p* < 0.05, ** *p* < 0.01, *** *p* < 0.001.

**Table 5:** Indirect effects in mediation model per group in Study 2.

Effect	Group	Estimate, Bootstrapped 95% CI
Mathematical Achievement	*noF&S/density*	**0.15 [0.06, 0.27]**
	*noF&S/speed*	**0.15 [0.05, 0.26]**
	*F&S/density*	**0.22 [0.12, 0.34]**
	*F&S/speed*	**0.24 [0.11, 0.39]**
Concept of Density	*noF&S/density*	0.04 [−0.01, 0.11]
	*noF&S/speed*	0.01 [−0.01, 0.05]
	*F&S/density*	0.01 [−0.03, 0.06]
	*F&S/speed*	0.06 [−0.04, 0.18]

Note. Bolded values denote significant effects.

## Data Availability

The data presented in this study are openly available in the Open Science Framework at https://osf.io/7ch48/. The lesson plans and further material (in German) are available upon request.
